# Is It Necessary to Add the Feedback Insufflation Time in Manikins? A Simulation Pilot Study

**DOI:** 10.3390/reports7030064

**Published:** 2024-08-01

**Authors:** Luis Castro-Alonso, Eloy Carracedo-Rodríguez, Martín Otero-Agra, Sheila Vázquez-Álvarez, Roberto Barcala-Furelos, María Fernández-Méndez

**Affiliations:** 1 Complexo Hospitalario of Pontevedra, Sergas, 36071 Pontevedra, Spain; lcastroalon@gmail.com; 2 REMOSS Research Group, Faculty of Education and Sports Sciences, University of Vigo, 36005 Pontevedra, Spain; carracedo54@gmail.com (E.C.-R.); sheilavzquez@gmail.com (S.V.-Á.); roberto.barcala@uvigo.es (R.B.-F.); mariajosefernandezmendez@gmail.com (M.F.-M.); 3 School of Nursing, University of Vigo, 36005 Pontevedra, Spain; 4 CLINURSID Research Group, Psychiatry, Radiology, Public Health, Nursing and Medicine Department, Universidade de Santiago de Compostela, 15782 Santiago de Compostela, Spain; 5 Simulation and Intensive Care Unit of Santiago (SICRUS) Research Group, Health Research Institute of Santiago, University Hospital of Santiago de Compostela-CHUS, 15706 Santiago de Compostela, Spain

**Keywords:** CPR, CPR training, first responders, bag-mask ventilation

## Abstract

(1) Objective: This study aimed to assess the evolution of the quality of ventilations of a group of rescuers after two training sessions by taking into account inspiration times. (2) Materials and Methods: A pilot simulation study was carried out with a sample of 10 lifeguard students. Two training sessions were held three weeks apart, in which CPR skills were trained by means of feedback tools. Participants performed three tests in pairs on a ResusciAnne QCPR^®^ manikin connected to SkillReporter QCPR software, namely one pre-training test and one test after each training session. CPR was performed in pairs for two minutes and began with five rescue breaths. (3) Results: One training session was enough to improve chest compression quality (T0: 48%; IQR 17–77/T1: 83%; IQR 59–88; *p* = 0.022/T2: 79%; IQR 64–92; *p* = 0.002). The quality of the ventilations increased progressively in each training session without reaching high-quality results (T0: 0%; IQR 0–0/T2: 15%; IQR 8–27; *p* = 0.011). (4) Conclusion: A two-session training program focused on inspiratory times achieved significant improvements in the quality of bag-mask ventilations performed by lifeguard students. Training focused on the insufflation time of ventilations and not only on the volume seems to be an important factor in improving the quality of ventilations.

## 1. Introduction

Rescuers play a fundamental role in the initial management of victims of cardiorespiratory arrest due to drowning. Because of the characteristics of the pathophysiological process, such victims require high-quality ventilatory treatment to rapidly reverse hypoxia [1]. For this reason, the people who must respond to this type of situation must be competent at performing cardiopulmonary resuscitation (CPR), depending on the level of rescue they provide, especially in the ventilatory support of the victims [2].

In recent years, the compression-only CPR technique for bystanders has been promoted [1]. This is because it is a technique that requires little or no prior training and has positive outcomes in the survival of cardiac arrest victims [1,3]. On the other hand, conventional CPR, especially when using bag-valve-mask ventilation, requires more training time [3]. However, in cases of cardiorespiratory arrest due to drowning attended by lifeguards, it should be the technique of choice due to the key factor of ventilations in the survival of these victims [2]. The compression-only CPR technique is a very interesting strategy for training the general population, but lifeguards must have specialized training in applying bag-valve-mask ventilations to perform high-quality CPR [4]. In this regard, recent evidence suggests that learning chest compressions is not difficult and does not require much time, but performing high-quality ventilations remains a challenge for training programs, as these are much more complex skills [5].

On the other hand, the European Resuscitation Council (ERC) 2021 guidelines no longer refer to the recommended volume of breaths (which was previously around 500–600 mL) [6]. Instead, they do highlight the importance of an inspiration time of approximately 1 s, along with observing chest rise and avoiding sudden inflations [7]. Sudden ventilations lead to complications such as increased intrathoracic pressure or a higher risk of bronchial aspiration [8,9,10,11,12]. Currently, feedback from training devices primarily focuses on the volume delivered, neglecting inspiratory times. As a result, most contemporary sham studies do not assess the quality of ventilation according to current ERC recommendations.

This study aimed to assess the enhancement of bag-mask ventilation quality by considering both volume and insufflation time following a training program that focused on the current recommendations for bag-mask ventilation.

## 2. Methods

### 2.1. Design

A pilot study was carried out using a quasi-experimental simulation design without a control group. The study took place between March and April 2022, lasting 3 weeks.

Through further analysis, an observational case-control study was also conducted using 381 ventilations performed during three simulation tests as a sample. This allowed for the evaluation of ventilations performed by study participants, categorized into three groups based on volume and insufflation time, with regard to the number of training sessions they underwent (participant exposure to training sessions: T0 = no training session; T1 = one training session; T2 = two training sessions).

### 2.2. Sample

To carry out this study, a non-probabilistic convenience sample of volunteers belonging to the Galician Civil Protection Corps who were undergoing training to become professional lifeguards was used. The inclusion criterion for participation was obtaining informed consent from those attending the course. The exclusion criteria were non-attendance to the training sessions or not having taken all the study tests. All 10 participants of the aforementioned course voluntarily agreed to participate in the study, and no volunteers were excluded. This study respected all the ethical principles of the Declaration of Helsinki.

In addition, for the observational study on ventilation, the ventilations performed by the participants were taken as a sample. The inclusion criterion for analyzing ventilations was that they had to be effective ventilations captured by the device providing the data, resulting in a final sample of 381 ventilations (147 at T0; 108 at T1; 126 at T2).

### 2.3. Measuring Devices

Participants’ training and test data collection were carried out using an adult Resusci Anne QCPR^®^ manikin of the Laerdal brand (Stavanger, Norway) that was connected to SkillReporter QCPR^®^ software of the same brand and configured by the 2021 ERC recommendations [7]. The software was used as a feedback method in the training sessions and enabled the CPR variables in the tests to be obtained (the participants did not receive any feedback during the tests). The volume of each ventilation was recorded through a checklist by a researcher at the time of its execution. The time of each ventilation was obtained through the metadata provided by the software, which accurately records the data (in milliseconds). Ventilations were performed using an adult-size The Bag II self-inflating bag from Laerdal (Stavanger, Norway) with a No. 5 mask and a disposable PEEP valve compatible with the bag.

### 2.4. Study Description

**Figure 1:**
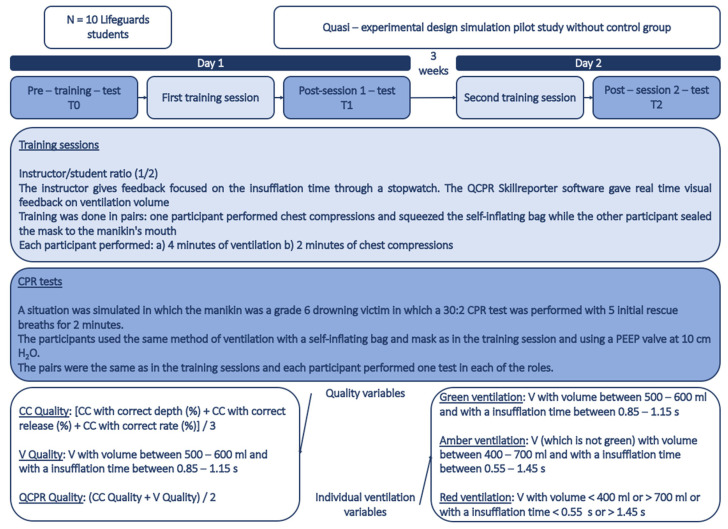
Flow-chart.

The description of the study is shown in Figure 1. The study began with a 2 min pre-training test (T0) of CPR in pairs. At the end of the tests, the participants attended the first training session, in which they performed a 4 min training in ventilations and a 2 min training in chest compressions. At the end, they took the post-session-1-test (T1). Three weeks later, the participants received the second training session, which had the same characteristics as the first one. They also took the post-session-2-test (T2) at the end.

The practical CPR training was conducted in pairs, with both participants performing both studied roles (Role A: chest compressions + squeezing the self-inflating bag/Role B: sealing the mask to the manikin’s mouth). The instructor-to-student ratio was 1:2. In addition to the feedback provided by the software for chest compressions and ventilations, the instructor provided feedback focused on the insufflation time, giving instructions aided by a stopwatch to achieve ventilation of approximately 1 s. The feedback provided consisted of measuring the duration of insufflation and, based on the recorded data from the ventilation, guiding the participant to adjust the duration in subsequent breaths.

The three tests were conducted in identical situations: a simulated drowning victim (manikin) using the adult 30:2 protocol preceded by 5 initial ventilations for 2 min. For this, participants used the bag and mask ventilation method for two rescuers and with the PEEP valve applying 10 cm H_2_O, according to ERC recommendations [13].

### 2.5. Calculated Variables

In addition to the variables provided by the SkillReporter QCPR^®^ software and the previously described milliseconds, ad-hoc variables were calculated using data obtained from these tools:

On the one hand, we have the calculated QCPR variables:

- Compression quality: [Compressions with correct depth (%) + Compressions with correct recoil (%) + Compressions with correct average rate (%)]/3;
- Ventilation quality: [Ventilations with a volume between 500–600 mL and inspiration time between 0.85 and 1.15 s (%)];
- CPR quality: (Compression quality + Ventilation quality)/2.

On the other hand, all ventilations from each test were analyzed individually and classified by color according to their volume and inspiration time. Ventilations categorized with green were those closest to the manual ventilation recommendations, those categorized with amber deviated slightly from these recommendations, and those categorized with red clearly diverged from the recommended guidelines. The ranges used for these categories were chosen arbitrarily based on deviations from the reference values for each variable (volume: 500–600 mL [6]/insufflation time: approximately 1 s [1]), while also considering acceptable parameters in mechanical ventilation programming [14]. The ranges for each color category are detailed below:

- Green ventilation: Ventilation meeting two conditions: (1) volume between 500 and 600 mL, and (2) insuflation time between 0.85 and 1.15 s;
- Amber ventilation: Ventilation meeting three conditions: (1) failing to meet one or none of the conditions for green ventilation, (2) volume between 400 and 700 mL, and (3) insufflation time between 0.55 and 1.45 s;
- Red ventilation: Ventilation meeting any of the following conditions: (1) volume < 400 mL, (2) volume > 700 mL, or (3) insufflation time < 0.55 s. (4) Insufflation time > 1.45 s.

### 2.6. Statistical Analysis

Data analysis was carried out with Microsoft Excel and IBM SPSS Statistics version 21 software. Qualitative variables were described through relative and absolute frequencies. The Chi-Square test was used for the comparisons between the different tests of the study and Cramer’s V test was used for the effect size. The following classification was used to categorize the effect size: 0.1–0.3 Small; 0.3–0.5 Medium; ≥0.5 Large. Quantitative variables were described through measures of central tendency (the median) and measures of dispersion (the interquartile range). For comparisons between the different study tests, the ANOVA test of repeated measures with Bonferroni correction was used for the variables that followed a normal distribution (effect size with Cohen’s d test) and the Friedman test for repeated measures with Bonferroni correction for variables that did not follow a normal distribution (effect size with Rosenthal’s r test). To categorize the effect size, the following classification was used: <0.2 Trivial; 0.2–0.5 Small; 0.5–0.8 Moderate; 0.8–1.3 Large; ≥1.3 Very large. A value of *p* = 0.05 was assigned for all analyses.

For the case–control design analysis of ventilations, percentages between cases and controls (red ventilation, amber ventilation, and green ventilation) were compared as a function of the participant’s exposure to training at the time of performing each ventilation (T0, T1, and T2) using the Chi-Square test and the Cramer’s V test for effect size. For pairwise comparisons, Bonferroni correction was used, adjusting the *p*-value to 0.0167 (0.05/3). The odds ratio was also calculated to assess the weight of exposure in the achievement or not of ventilation with certain characteristics.

## 3. Results

### 3.1. Demographic Variables of the Lifeguard Students

The results of the demographic variables are shown in Table 1. All participants had prior training, 50% having received their last CPR training less than a year previously.

### 3.2. CPR Variables

The results of the CPR variables are shown in Table 2. No significant differences were observed in terms of the average depth of compressions. However, the average rate obtained significantly lower values in the post-session 2 test (T2: median 113; IQR 108–120; *p* = 0.020) compared to the pre-training test (T0: median 122; IQR 116–127).

Regarding ventilation variables, significantly higher values were observed in no-flow time after both sessions (T1: median 10%; IQR 8–10; *p* = 0.016/T2: median 9%; IQR 9–10; *p* = 0.004) compared to the pre-training test (T0: median 6%: IQR 5–7). Significantly lower values were observed in the number of ventilations in both post-training tests (T1: median 13; IQR 11–13; *p* = 0.004/T2: median 13; IQR 12–13; *p* = 0.016) compared to the pre-training test (T0: median 15; IQR 15–15). No significant differences were observed in terms of the percentage of effective ventilations. Significantly higher values were observed in the mean of ventilation volume in the pre-training test (T0: median 639 mL; IQR 593–717; *p* = 0.003) compared to the first post-training test (T1: median 452 mL; IQR 317–519). The mean inspiration time had a significant increase after the training sessions compared to the pre-test (T0: 0.48 s; IQR 0.41–0.59/T1: 0.91 s; IQR 0.73–1.21; *p* = 0.030/T2: 0.94 s; IQR 0.82–1.03; *p* < 0.001). Compression quality was also significantly improved after training compared to the pre-test (T0: 48%; IQR 17–77/T1: 83%; IQR 59–88; *p* = 0.022/T2: 79%; IQR 64–92; *p* = 0.002). With regards to the quality of ventilation, significant improvements were observed only in T2 (15%; IQR 8–27) when compared to T0 (0%; IQR 0–0; *p* = 0.011), with no significant differences observed in other measurements Regarding the quality of CPR based on volume and inspiration time, significantly higher results were observed after the two training sessions compared to the pre-test (T0: 24%; IQR 9–38/T1: 43%; IQR 30–50; *p* = 0.011/T2: 49%; IQR 40–58; *p* = 0.011).

### 3.3. Case-Control Analysis

**Figure 2:**
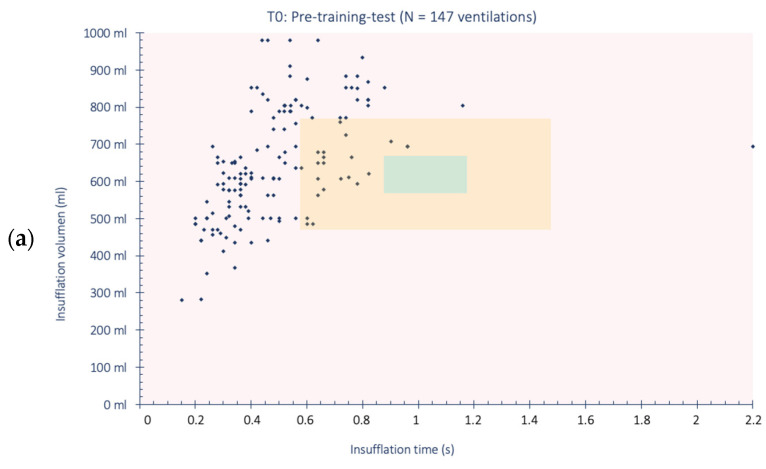

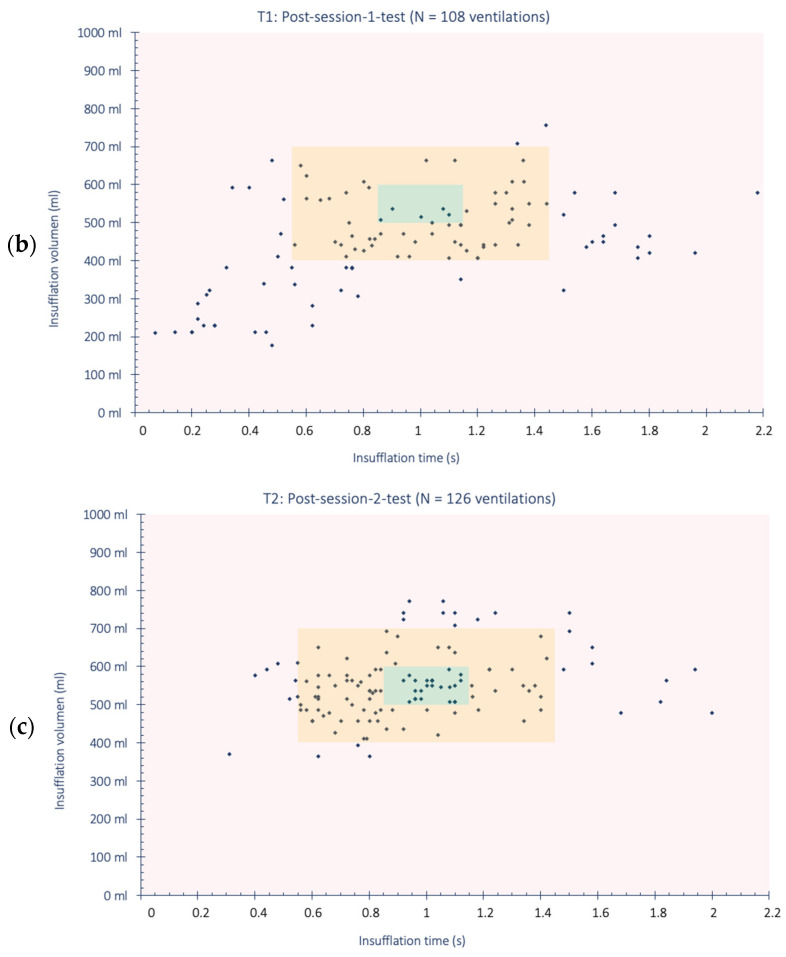
Analysis of all ventilations individually according to the test: (**a**) T0 ventilations; (**b**) T1 ventilations; (**c**) T2 ventilations.

The description of the individual breaths is shown in Figure 2. The volume and inspiratory time of a total of 381 breaths were analyzed (147 in T0; 108 in T1; 126 in T2). Regarding the ventilations that were framed in the green square, a greater number of ventilations was observed in T1 compared to the pre-test. The same occurs in T2 compared to T1 and with the pre-test. The description of the type of ventilation performed by each participant is shown in the Supplementary Figure S1.

Regarding the case–control analysis (Table 3), an odds ratio of 1.86 (1.44–2.39) was observed for performing green ventilation and an odds ratio of 3.10 (2.24–4.28) for performing amber ventilations after two training sessions, taking as a reference the performance of red ventilation without training.

## 4. Discussion

The objective of this study was to assess the improvement of the quality of rescuers’ ventilations after a training program that focused on compliance with current recommendations. The main finding of this study was the positive evolution of ventilation quality, mainly concerning the insufflation time, which showed significant improvements based on these recommendations.

It was observed in this study that despite the major emphasis on ventilations, two short training sessions with instructor-led practical advice and real-time feedback were sufficient to obtain high-quality chest compressions. These results coincide with those obtained in other studies [15,16].

On one hand, this study observed an increase in hands-free time and a decrease in compression time after both training sessions, dedicating more time to ventilation administration. This led to a significant improvement in the quality of ventilations, consistently staying within the 10 s limit that the ERC recommends not to exceed regarding compression interruptions [7]. Additionally, a decrease in the total number of ventilations was observed. This was because, in the pre-training test, inspiratory times were shorter, allowing for a greater number of ventilations compared to post-training tests, where fewer ventilations were observed but of higher quality. These findings are considered beneficial as they succeeded in improving the quality of ventilation, which is crucial in the context of drowning victim care.

On the other hand, although the training tools record the inspiration times of the breaths, they are not shown in real-time, thus hindering the training according to the most current ERC recommendations for insufflations of approximately 1 s [7]. Therefore, the vast majority of simulation studies in the scientific literature focus on assessing parameters such as volume, frequency, or ventilation effectiveness, without taking these inspiratory values into account [17,18,19].

In the initial test of this study, ventilations were observed with much shorter inspiration times in relation to current guidelines. These values coincide with those obtained by Adelborg et al. [20]. However, it has been observed that ventilations with excessive inflation volume and pressure due to a short inspiratory time can lead to a significant increase in intrathoracic pressure, possibly causing hemodynamic changes [8,9,10]. In addition, this type of ventilation may cause gastric insufflation, which increases the risk of regurgitation and bronchial aspiration [11,12].

Despite not having real-time feedback methods for inspiratory values, significant improvements were observed even by practicing using these parameters inaccurately, as an average inspiration time of 0.94 s was obtained after the two sessions. However, having surely been influenced by the deficiencies of the mannequins, after two training sessions only a median quality of ventilation of 15% was observed, with 19% of ventilations having the correct volume and inflation time. Therefore, despite having shown some improvement, this training program has not been enough to achieve high-quality ventilation.

This study jointly evaluated volume and inspiration times. To date, no study has been found that evaluates both parameters together. The results indicated that performing ventilations with both appropriate volume and inspiration time is more complex than administering ventilations with correct volumes alone. However, a significant improvement in ventilations in the green category has been observed, increasing from 0% in the pre-training test to 19% after both training sessions. Furthermore, if we combine the green and amber categories, we can observe that in the pre-training test, 15% of ventilations fell within these intervals, compared to 78% after both training sessions.

In this regard, the case–control study of the 381 ventilations performed during the simulation tests showed that there may be an association between training based on insufflation time feedback and the performance of ventilations that are closer to the volume and ventilation time parameters recommended by the ERC [1].

In short, increasing the number of sessions could be sufficient to achieve high-quality ventilation. Furthermore, having real-time feedback on inspiratory values would be a fundamental tool that would allow more efficient training sessions to achieve high-quality ventilation more quickly, as in this study the feedback of the insufflation time during training was provided by the instructor using a stopwatch. The possibility of a high-fidelity manikin providing live insufflation time would exponentially improve the accuracy of the feedback and therefore its quality.

This study has some limitations. It was carried out in a simulated environment, so the results cannot be directly extrapolated to clinical practice. Likewise, the sample size of this pilot study was limited, in addition to not presenting a control group, which constrains the strength of the evidence.

## 5. Conclusions

A two-session ventilation training program focused on inspiratory times achieved significant improvements in the quality of bag-mask ventilations performed by lifeguard students. Emphasizing insufflation time rather than solely focusing on volume appears to be a crucial factor in enhancing ventilation quality. CPR training should account for the recommendation to avoid abrupt breaths and to aim for an inflation time of approximately 1 s.

## Figures and Tables

**Table 1:** Demographic variables (N = 10).

**Quantitative Variables**		**Median**	**IQR**
Age (in years)		21	(20–22)
Weight (in kg)		76	(68–94)
Height (in cm)		179	(174–181)
**Qualitative variables**		**N**	**(%)**
Sex			
	Women	4	(40%)
	Men	6	(60%)
Previous CPR training			
	<1 year	5	(50%)
	>1 year	5	(50%)

IQR: Interquartile range (Q1–Q3); N: Absolute frequency. (%): Relative frequency.

**Table 2:** CPR variables (N = 10).

	T0		T1		T2		*p*-Value
	Median	IQR	Median	IQR	Median	IQR	
CC variables							
Mean depth (mm)	56	(52–61)	53	(50–55)	54	(50–56)	NS
Mean rate (CC/min)	122	(116–127)	115	(106–120)	113	(108–120)	T0 vs. T1 = 0.09 T0 vs. T2 = 0.020 † (1.12) T1 vs. T2 = 1.00
V variables							
No flow time (s)	6	(5–7)	10	(8–10)	9	(9–10)	T0 vs. T1 = 0.016 * (0.88) T0 vs. T2 = 0.004 * (1.03) T1 vs. T2 = 1.00
Number of total V	15	(15–15)	13	(11–13)	13	(12–13)	T0 vs. T1 = 0.004 * (1.03) T0 vs. T2 = 0.016 * (0.88) T1 vs. T2 = 1.00
Effective V (%)	100	(100–100)	100	(78–100)	100	(100–100)	NS
Mean volume (mL)	639	(593–717)	452	(317–519)	531	(488–618)	T0 vs. T1 = 0.003 † (2.07) T0 vs. T2 = 0.06 T1 vs. T2 = 0.20
Mean insufflation time (s)	0.48	(0.41–0.59)	0.91	(0.73–1.21)	0.94	(0.82–1.03)	T0 vs. T1 = 0.030 † (1.57) T0 vs. T2 < 0.001 † (3.17) T1 vs. T2 = 1.00
Quality variables							
CC quality (%)	48	(17–77)	83	(59–88)	79	(64–92)	T0 vs. T1 = 0.022 * (0.85) T0 vs. T2 = 0.002 * (1.06) T1 vs. T2 = 1.00
V quality (%)	0	(0–0)	0	(0–11)	15	(8–27)	T0 vs. T1 = 0.79 T0 vs. T2 = 0.011 * (0.92) T1 vs. T2 = 0.22
CPR quality (%)	24	(9–38)	43	(30–50)	49	(40–58)	T0 vs. T1 = 0.011 † (1.31) T0 vs. T2 = 0.011 † (1.68) T1 vs. T2 = 0.71

NS: Not significant; CC: Chest compressions; V: Ventilations; CPR: Cardiopulmonary resuscitation; T0: Pre-training-test. T1: Post-session-1-test. T2: Post-session-2-test. IQR: Interquartile range (Q1–Q3); * Friedman test with Bonferroni correction (*p* = 0.05). In brackets, effect size with Rosenthal’s r test; † ANOVA test with Bonferroni correction (*p* = 0.05). In brackets, effect size with Cohen’s d test. Effect size classification: <0.2 Trivial; 0.2–0.5 Small; 0.5–0.8 Moderate; 0.8–1.3 Large; ≥1.3 Very large.

**Table 3:** Case–control analysis (based on volume and insufflation time categories) of ventilations according to the time of exposure (training sessions performed).

	Green Ventilation (N = 30)	Amber Ventilation (N = 149)	
	N (%)	N (%)	
T0 (No training with feedback insufflation time)	0 (0%)	22 (15%)	*p* = 0.005 (0.24) T0 vs. T1 = 0.12 T0 vs. T2 = 0.009 * (0.24) OR: 1.32 (1.18–1.48) T1 vs. T2 = 0.03
T1 (one session with feedback insufflation time)	6 (20%)	53 (35%)	
T2 (two sessions with feedback insufflation time)	24 (80%)	74 (50%)	
	Green ventilation (N = 30)	Red ventilation (N = 202)	
	N (%)	N (%)	
T0 (No training with feedback insufflation time)	0 (0%)	125 (62%)	*p* < 0.001 (0.55) T0 vs. T1 < 0.001 * (0.28) OR: 1.12 (1.02–1.23) T0 vs. T2 < 0.001 * (0.61) OR: 1.86 (1.44–2.39) T1 vs. T2 < 0.001 * (0.39) OR: 1.66 (1.27–2.16)
T1 (one session with feedback insufflation time)	6 (20%)	49 (24%)	
T2 (two sessions with feedback insufflation time)	24 (80%)	28 (14%)	
	Amber ventilation (N = 149)	Red ventilation (N = 202)	
	N (%)	N (%)	
T0 (No training with feedback insufflation time)	22 (15%)	125 (62%)	*p* < 0.001 (0.50) T0 vs. T1 < 0.001 * (0.40) OR: 1.77 (1.43–2.19) T0 vs. T2 < 0.001 * (0.58) OR: 3.10 (2.24–4.28) T1 vs. T2 = 0.002 * (0.21) OR: 1.75 (1.20–2.55)
T1 (one session with feedback insufflation time)	53 (35%)	49 (24%)	
T2 (two sessions with feedback insufflation time)	74 (50%)	28 (14%)	

N: Absolute frequency. (%): Relative frequency. T0: Pre-training-test. T1: Post-session-1-test. T2: Post-session-2-test. * Chi-Square test (*p* = 0.05) with Bonferroni correction for comparisons between tests (*p* = 0.0167). In pairs, Effect Size with Cramer’s V test. OR: Odds Ratio, with 95% confidence intervals in pairs.

## Data Availability

The original contributions presented in this study are included in the article. For further inquires, please contact the corresponding author.

## Supplementary Materials

The following supporting information can be downloaded at: https://www.mdpi.com/article/10.3390/reports7030064/s1, Figure S1: Description of the type of ventilations in each test for each of the study participants.
