# Supplementary material for: Is It Necessary to Add the Feedback Insufflation Time in Manikins? A Simulation Pilot Study
Source: Reports (MDPI). 2024 Aug 1;7(3):64. doi: 10.3390/reports7030064 (PMC12225349; doi:10.3390/reports7030064)
Supplement: Supplementary file 1 [file reports-07-00064-s001.zip › reports-3115323-supplementary.pdf]

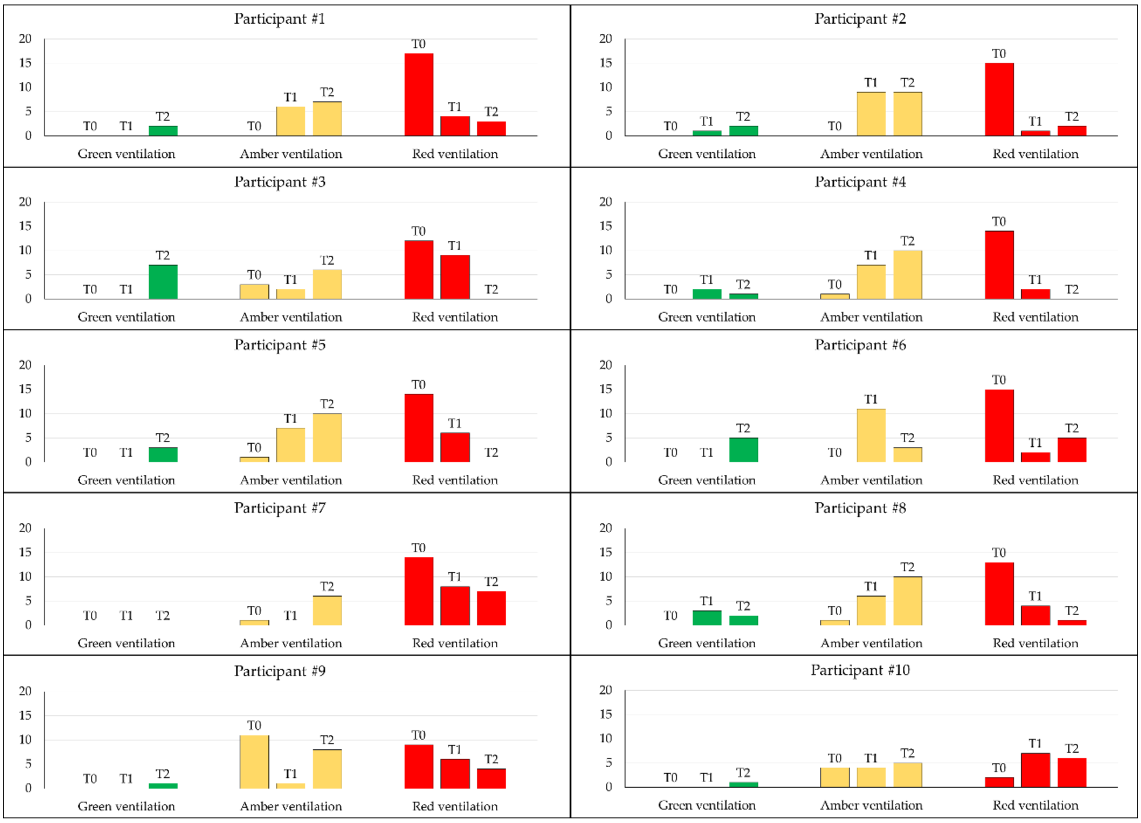

Supplementary Figure S1: Description of the type of ventilations in each test for each of the study participants
